# Supplementary material for: Pollen Exposure and Cardiopulmonary Health Impacts in Adelaide, South Australia
Source: Int J Environ Res Public Health. 2022 Jul 26;19(15):9093. doi: 10.3390/ijerph19159093 (PMC9331296; doi:10.3390/ijerph19159093)
Supplement: Supplementary file 1 [file ijerph-19-09093-s001.zip › ijerph-1801899-supplementary.pdf]

Supplementary tables

Table S1: Short-term incidence rate ratios (IRRs) in adults for cardiovascular-related hospital admissions (H), emergency department presentations (E) and ambulance callouts (A) and pollen (Grasses, Weeds, Trees) by month.

Cardiovascular health outcomes in adults (18 years and older)										
		Grasses			Trees			Weeds		
		A	E	H	A	E	H	A	E	H
IRR	Jan	1.02	0.95	1.00	1.04	1.00	0.95	0.97	1.03	1.04
95% CI		0.85-1.23	0.77-1.17	0.85-1.17	0.92-1.17	0.87-1.15	0.86-1.04	0.81-1.15	0.85-1.26	0.91-1.18
p		0.81	0.62	0.99	0.54	0.98	0.29	0.69	0.77	0.59
IRR	Feb	0.91	0.86	0.98	1.04	1.01	1.00	1.05	1.12	1.01
95% CI		0.78-1.05	0.73-1.02	0.87-1.10	0.93-1.16	0.90-1.14	0.91-1.10	0.90-1.23	0.96-1.32	0.91-1.12
p		0.18	0.08	0.71	0.49	0.85	1.00	0.50	0.15	0.89
IRR	Mar	**1.16**	1.05	**1.11**	1.02	1.06	0.99	0.88	0.92	0.92
95% CI		**1.01-1.33**	0.90-1.22	**1.00-1.24**	0.92-1.12	0.95-1.18	0.91-1.07	0.77-1.01	0.80-1.06	0.83-1.01
p		**0.03**	0.55	**0.04**	0.74	0.29	0.72	0.07	0.26	0.08
IRR	Apr	1.01	1.04	1.10	0.99	1.00	1.02	1.01	0.98	0.94
95% CI		0.89-1.14	0.89-1.21	0.98-1.23	0.89-1.10	0.89-1.13	0.94-1.10	0.90-1.13	0.86-1.12	0.85-1.04
p		0.88	0.62	0.10	0.84	0.98	0.72	0.91	0.78	0.21
IRR	May	1.09	**1.14**	**1.14**	0.97	0.99	1.03	0.99	0.94	0.95
95% CI		1.00-1.19	**1.01-1.29**	**1.04-1.25**	0.91-1.04	0.91-1.08	0.97-1.09	0.91-1.07	0.85-1.05	0.88-1.03
p		0.06	**0.03**	**0.01**	0.42	0.80	0.34	0.78	0.26	0.21
IRR	Jun	0.90	1.16	1.19	1.03	1.03	1.03	1.03	0.97	0.95
95% CI		0.78-1.04	0.96-1.40	1.02-1.38	0.98-1.08	0.97-1.10	0.98-1.07	0.95-1.11	0.86-1.08	0.87-1.04
p		0.16	0.12	0.02	0.27	0.31	0.24	0.46	0.56	0.26
IRR	Jul	0.94	0.71	0.86	**1.05**	**1.09**	1.03	0.87	0.86	0.90
95% CI		0.73-1.20	0.52-0.98	0.67-1.09	**1.00-1.10**	**1.03-1.16**	0.99-1.08	0.77-0.99	0.70-1.06	0.76-1.07
p		0.60	0.04	0.21	**0.04**	**0.00**	0.16	0.04	0.15	0.22
IRR	Aug	0.88	0.85	0.90	1.02	1.03	1.00	0.94	1.03	0.96
95% CI		0.78- 1.01	0.72-1.01	0.79-1.02	0.97-1.07	0.97-1.10	0.95-1.04	0.84-1.05	0.89-1.19	0.85-1.08
p		0.07	0.06	0.11	0.45	0.32	0.86	0.26	0.69	0.49
IRR	Sep	0.95	0.92	1.05	1.00	1.02	0.96	1.02	1.06	0.99
95% CI		0.87-1.04	0.81-1.05	0.95-1.15	0.94-1.06	0.95-1.10	0.91-1.02	0.94-1.11	0.96-1.18	0.91-1.08
p		0.30	0.20	0.36	0.99	0.59	0.20	0.61	0.26	0.82
IRR	Oct	**1.14**	0.95	0.99	0.97	1.03	0.95	0.92	1.04	1.03
95% CI		**1.01-1.27**	0.82-1.10	0.88-1.11	0.89-1.05	0.93-1.13	0.88-1.02	0.85-1.00	0.94-1.16	0.95-1.12
p		**0.03**	0.47	0.86	0.41	0.61	0.16	0.06	0.45	0.46
IRR	Nov	1.05	0.81	1.08	0.98	**1.25**	0.95	1.00	0.95	0.94
95% CI		0.92-1.19	0.69-0.96	0.95-1.23	0.89-1.06	**1.12-1.40**	0.88-1.04	0.90-1.12	0.83-1.09	0.85-1.04
p		0.46	0.01	0.25	0.58	**0.00**	0.26	0.97	0.46	0.22
IRR	Dec	1.11	0.92	1.09	0.94	**1.19**	0.98	1.02	0.89	0.91
95% CI		0.97-1.27	0.77-1.09	0.96-1.24	0.84-1.05	**1.03-1.38**	0.90-1.08	0.91-1.13	0.78-1.01	0.83-0.99
p		0.12	0.33	0.19	0.28	**0.02**	0.73	0.75	0.06	0.03

Table S2: Short-term incidence rate ratios (IRRs) in adults for lower respiratory-related hospital admissions (H), emergency department presentations (E) and pollen (Grasses, Weeds, Trees) by month.

		Pollen and lower respiratory health outcomes in adults					
		Grasses		Trees		Weeds	
		E	H	E	H	E	H
IRR	Jan	0.85	0.96	1.05	0.91	1.06	1.33
95% CI		0.57-1.27	0.60-1.52	0.82-1.35	0.69-1.20	0.73-1.54	0.88-2.00
p		0.43	0.85	0.70	0.51	0.75	0.18
IRR	Feb	1.06	1.29	1.12	1.00	0.88	0.96
95% CI		0.76-1.48	0.87-1.91	0.88-1.41	0.75-1.34	0.63-1.23	0.66-1.41
p		0.73	0.20	0.35	0.97	0.46	0.84
IRR	Mar	1.09	1.20	1.09	1.18	0.90	0.93
95% CI		0.82-1.47	0.85-1.70	0.88-1.35	0.92-1.51	0.68-1.18	0.67-1.28
p		0.55	0.30	0.42	0.21	0.44	0.64
IRR	Apr	1.02	1.06	0.99	0.94	0.99	1.09
95% CI		0.78-1.33	0.74-1.50	0.80-1.24	0.73-1.21	0.77-1.25	0.79-1.49
p		0.89	0.77	0.95	0.63	0.91	0.60
IRR	May	0.97	0.97	1.06	1.12	0.94	0.92
95% CI		0.80-1.17	0.76-1.24	0.93-1.22	0.95-1.31	0.79-1.11	0.74-1.13
p		0.75	0.81	0.39	0.19	0.44	0.42
IRR	Jun	0.75	1.36	1.02	1.09	1.17	0.87
95% CI		0.57-0.99	0.96-1.91	0.93-1.11	0.98-1.22	1.00-1.37	0.71-1.07
p		0.04	0.08	0.70	0.11	0.05	0.20
IRR	Jul	0.74	0.90	1.07	1.06	1.02	0.92
95% CI		0.49-1.10	0.53-1.54	0.99-1.17	0.96-1.18	0.81-1.29	0.68-1.25
p		0.14	0.71	0.09	0.25	0.85	0.60
IRR	Aug	1.05	1.06	1.01	1.03	0.95	1.15
95% CI		0.86-1.29	0.80-1.40	0.92-1.10	0.92-1.14	0.80-1.13	0.88-1.50
p		0.63	0.69	0.89	0.63	0.55	0.32
IRR	Sep	1.02	1.12	1.05	1.10	0.95	1.01
95% CI		0.87-1.19	0.91-1.36	0.95-1.16	0.97-1.25	0.83-1.08	0.84-1.21
p		0.83	0.28	0.31	0.12	0.45	0.92
IRR	Oct	0.93	1.05	1.10	1.16	0.97	0.96
95% CI		0.76-1.14	0.80-1.36	0.96-1.26	0.98-1.38	0.83-1.12	0.80-1.16
p		0.49	0.73	0.18	0.09	0.66	0.71
IRR	Nov	1.04	1.02	1.04	**1.29**	0.96	0.83
95% CI		0.80-1.36	0.74-1.43	0.87-1.23	**1.04-1.59**	0.76-1.19	0.63-1.10
p		0.76	0.88	0.67	**0.02**	0.69	0.20
IRR	Dec	1.09	1.05	0.88	1.03	1.12	1.09
95% CI		0.82-1.45	0.74-1.50	0.70-1.11	0.79-1.33	0.89-1.40	0.85-1.40
p		0.55	0.77	0.27	0.84	0.34	0.51

Table S3: Short-term incidence rate ratios (IRRs) in children for lower respiratory-related hospital admissions (H), emergency department presentations (E) and pollen (Grasses, Weeds, Trees) by month.

		Pollen and lower respiratory health outcomes in children					
		Grasses		Trees		Weeds	
		E	H	E	H	E	H
IRR	Jan	1.33	1.45	1.04	0.81	0.64	0.84
95% CI		0.85-2.08	0.79-2.66	0.80-1.36	0.58-1.13	0.42-0.99	0.49-1.45
p		0.20	0.23	0.76	0.21	0.04	0.54
IRR	Feb	0.70	0.85	0.95	1.20	**1.42**	0.97
95% CI		0.53-0.92	0.58-1.23	0.77-1.18	0.89-1.63	**1.07-1.90**	0.66-1.43
p		0.01	0.38	0.64	0.24	**0.02**	0.89
IRR	Mar	0.78	1.05	1.08	1.08	1.15	0.86
95% CI		0.59-1.02	0.72-1.52	0.89-1.30	0.82-1.41	0.90-1.48	0.61-1.21
p		0.07	0.81	0.43	0.59	0.27	0.38
IRR	Apr	0.84	0.84	0.97	1.04	1.19	1.10
95% CI		0.67-1.06	0.61-1.15	0.81-1.17	0.81-1.33	0.96-1.47	0.82-1.49
p		0.14	0.27	0.77	0.75	0.12	0.51
IRR	May	1.01	1.05	0.91	0.86	1.08	1.05
95% CI		0.89-1.16	0.87-1.27	0.83-1.00	0.76-0.98	0.95-1.21	0.89-1.24
p		0.84	0.60	0.06	0.02	0.24	0.55
IRR	Jun	1.00	0.92	1.02	1.01	1.01	0.97
LCL		0.85-1.19	0.73-1.17	0.96-1.07	0.93-1.09	0.91-1.11	0.84-1.12
p		0.96	0.52	0.57	0.85	0.91	0.70
IRR	Jul	0.80	0.67	1.03	**1.08**	1.08	1.10
95% CI		0.62-1.03	0.47-0.96	0.98-1.09	**1.01-1.17**	0.93-1.26	0.89-1.35
p		0.08	0.03	0.28	**0.04**	0.30	0.38
IRR	Aug	0.92	0.90	0.99	1.04	1.08	1.20
95% CI		0.79-1.07	0.73-1.12	0.93-1.05	0.96-1.12	0.95-1.24	0.99=1.45
p		0.28	0.36	0.77	0.37	0.25	0.07
IRR	Sep	0.87	0.97	1.06	**1.12**	1.01	0.99
95% CI		0.76-0.99	0.81-1.17	0.99-1.15	**1.01-1.24**	0.90-1.12	0.84-1.16
p		0.03	0.79	0.10	**0.03**	0.90	0.89
IRR	Oct	0.84	0.90	0.97	1.07	1.06	1.06
95% CI		0.70-1.01	0.70-1.16	0.87-1.09	0.91-1.24	0.93-1.21	0.88-1.26
p		0.06	0.42	0.64	0.41	0.39	0.56
IRR	Nov	0.97	0.84	0.93	0.99	1.04	1.24
LCL		0.76-1.22	0.59-1.19	0.80-1.09	0.80-1.22	0.85-1.26	0.94-1.64
p		0.78	0.33	0.37	0.90	0.72	0.12
IRR	Dec	0.97	1.05	1.08	1.13	0.92	0.96
LCL		0.72-1.30	0.68-1.62	0.85-1.37	0.83-1.53	0.74-1.16	0.71-1.29
p		0.82	0.81	0.51	0.45	0.49	0.77

Table S4: Short-term incidence rate ratios (IRRs) in adults for COPD-related hospital admissions (H), emergency department presentations (E) and ambulance callouts (A) and pollen (Grasses, Weeds, Trees) by month.

		Pollen and COPD health outcomes								
		Grasses			Trees			Weeds		
		A	E	H	A	E	H	A	E	H
IRR	Jan	1.07	1.22	1.04	1.09	0.97	1.02	0.90	0.89	1.02
95%CI		0.69-1.65	0.93-1.61	0.82-1.30	0.81-1.46	0.81-1.16	0.89-1.17	0.59-1.38	0.69-1.15	0.83-1.25
p		0.76	0.16	0.77	0.57	0.77	0.78	0.63	0.38	0.86
IRR	Feb	0.96	1.07	1.17	0.97	1.03	1.04	1.07	0.96	0.95
95%CI		0.69-1.35	0.86-1.34	0.98-1.39	0.73-1.29	0.87-1.21	0.91-1.20	0.73-1.56	0.77-1.20	0.80-1.12
p		0.84	0.55	0.08	0.84	0.76	0.55	0.73	0.73	0.53
IRR	Mar	0.98	1.11	1.13	1.18	1.03	1.03	0.84	0.94	0.99
95%CI		0.71-1.37	0.90-1.36	0.96-1.33	0.91-1.52	0.89-1.20	0.91-1.16	0.61-1.18	0.77-1.14	0.84-1.15
p		0.93	0.34	0.14	0.22	0.66	0.67	0.32	0.50	0.86
IRR	Apr	1.30	1.06	0.99	1.15	1.08	1.06	0.67	0.90	1.04
95%CI		0.95-1.77	0.88-1.28	0.84-1.17	0.88-1.49	0.93-1.27	0.94-1.20	0.50-0.89	0.75-1.07	0.89-1.20
p		0.10	0.53	0.93	0.31	0.31	0.36	0.01	0.22	0.64
IRR	May	**1.29**	1.01	1.05	0.96	0.97	1.00	0.76	0.97	1.00
95%CI		**1.04-1.58**	0.88-1.17	0.93-1.19	0.82-1.13	0.88-1.07	0.92-1.09	0.63-0.92	0.85-1.10	0.90-1.11
p		**0.02**	0.84	0.42	0.62	0.57	0.97	0.00	0.61	0.98
IRR	Jun	1.25	1.12	1.00	0.90	0.97	1.00	0.87	0.88	0.98
95%CI		0.90-1.74	0.91-1.39	0.83-1.20	0.81-1.01	0.91-1.04	0.95-1.06	0.73-1.03	0.78-1.00	0.88-1.10
p		0.18	0.28	0.98	0.08	0.35	0.88	0.11	0.05	0.78
IRR	Jul	0.84	0.90	0.90	1.03	1.00	1.03	0.86	1.03	0.98
95%CI		0.47-1.51	0.65-1.23	0.68-1.19	0.93-1.15	0.93-1.07	0.97-1.09	0.65-1.13	0.85-1.24	0.83-1.16
p		0.57	0.50	0.45	0.56	0.95	0.33	0.28	0.76	0.84
IRR	Aug	0.98	0.80	0.95	0.99	1.00	1.02	1.13	1.03	1.04
95%CI		0.73-1.32	0.67-0.96	0.82-1.10	0.88-1.10	0.94-1.07	0.97-1.08	0.89-1.42	0.89-1.19	0.91-1.18
p		0.89	0.02	0.52	0.81	0.92	0.44	0.31	0.71	0.58
IRR	Sep	1.07	1.00	0.93	1.02	1.04	1.06	0.91	0.94	1.02
95%CI		0.87-1.30	0.88-1.14	0.84-1.04	0.90-1.16	0.96-1.13	0.99-1.13	0.76-1.09	0.84-1.05	0.93-1.12
p		0.54	0.98	0.20	0.77	0.32	0.10	0.30	0.28	0.67
IRR	Oct	1.09	1.11	0.96	1.02	0.98	1.04	0.91	0.91	0.99
95%CI		0.84-1.42	0.94-1.32	0.84-1.11	0.85-1.23	0.88-1.09	0.95-1.13	0.74-1.11	0.80-1.02	0.90-1.10
p		0.52	0.21	0.58	0.81	0.73	0.44	0.35	0.11	0.88
IRR	Nov	0.85	1.09	1.12	1.04	0.90	0.92	1.14	1.04	1.03
95%CI		0.63-1.13	0.89-1.33	0.94-1.33	0.85-1.27	0.79-1.03	0.82-1.02	0.89-1.47	0.88-1.23	0.90-1.18
p		0.26	0.42	0.21	0.71	0.13	0.12	0.30	0.64	0.67
IRR	Dec	0.97	1.17	1.01	1.03	0.85	1.00	1.08	1.05	1.05
95%CI		0.72-1.32	0.94-1.45	0.85-1.21	0.81-1.33	0.71-1.01	0.88-1.14	0.83-1.41	0.89-1.24	0.92-1.19
p		0.86	0.15	0.90	0.79	0.06	0.97	0.56	0.54	0.49
